# Exploring undergraduate nursing student interactions with virtual patients to develop ‘non-technical skills’ through case study methodology

**DOI:** 10.1186/s41077-019-0088-7

**Published:** 2019-02-13

**Authors:** Monica Peddle, Margaret Bearman, Lisa Mckenna, Debra Nestel

**Affiliations:** 1 School of Nursing and Midwifery, College of Science Health and Engineering, La Trobe University, Melbourne, Australia; 2 Centre for Research in Assessment and Digital Learning (CRADLE), Deakin University, Melbourne, Australia; 3 Monash Institute for Health and Clinical Education, Faculty of Medicine, Nursing and Health Sciences, Monash University, Melbourne, Australia

**Keywords:** Virtual patient, Simulation, Non-technical skills

## Abstract

**Background:**

Virtual patients are a recent addition to the educational arsenal to develop non-technical skills in undergraduate health professionals. The Virtual Simulated Patient Resource (www.vspr.net.au) is a web-based resource that uses branching, narrative virtual patients to develop knowledge, attitude and practice of all categories of non-technical skills in undergraduate health professionals. However, there is limited literature exploring how the interaction with a virtual patient influences the development of knowledge, attitude and practice of non-technical skills in undergraduate nursing students.

**Methods:**

An intrinsic case study method, using focus groups and individual interviews, enabled exploration of the experience of undergraduate nursing students when interacting with a virtual patient to develop non-technical skills. Purposive sampling identified participants to address the research question. Framework analysis supported by a codebook enabled deductive and inductive data analysis.

**Results:**

Forty-five first-year and 31 third-year students consented to participate. Findings indicated that the different years interacted differently with the virtual patients. Four themes were recognised in the data: how the virtual patients enabled learning non-technical skills, learning surrounding the virtual patient encounter, changing the way students perceive practice and potential limitations to learning.

**Conclusions:**

Interactions with virtual patients influence learning knowledge, attitudes and practice of non-technical skills in undergraduate nursing students via authenticity in the virtual patient interaction, socialisation to the professional role, vicarious learning and learning by making mistakes. Potential limitations to learning from virtual patient interactions include fear, overconfidence, groupthink and confusion. To manage limitations to learning, facilitation approaches, opportunities for reflection, constructive feedback and debriefing may be key. This study demonstrates learning non-technical skills via interactions with virtual patients can change the way students perceive practice, with learning transferable to the clinical setting to support safe and competent patient care.

**Electronic supplementary material:**

The online version of this article (10.1186/s41077-019-0088-7) contains supplementary material, which is available to authorized users.

## Introduction

Virtual patients (VPs) are defined as “interactive computer simulations of real-life clinical scenarios for the purpose of healthcare and medical training, education, or assessment” [1]. Traditionally, VPs have been used to develop diagnostic and clinical reasoning in medical and nursing students [2, 3]. Advances in technology and educational methodology have supported the evolution of VPs to address wider areas of learning, including “non-technical skills” (NTS). Interactions with VPs are suggested to be effective in developing categories of NTS including communication, teamwork and decision-making across all health professions [4]. However, there are limited publications exploring how the experience of interacting with VPs influences students’ learning knowledge, skill, attitudes and practise of NTS. Understanding student learning experiences is important to ensure learning activities facilitate students’ academic development.

An integrative review identified features of interactions with VPs in developing NTS in undergraduate health professionals. Three themes were reported: socialisation to the professional role, transfer of learning to the clinical setting and authenticity [4]. However, studies included in the review only addressed isolated categories of NTS. No publication addressed the full suite of NTS categories of communication, situation awareness, teamwork, leadership, decision-making, coping with stress or managing fatigue. There is debate regarding the use of the term non-technical skills to describe these complex behaviours identified as critical components of safe and competent practice for health professionals [5]. We use the term in this paper whilst acknowledging its limitations.

The purpose of this study was to explore experiences of undergraduate nursing students when interacting with VPs designed to develop a broad range of NTS such as communication, situation awareness, teamwork, leadership, decision-making, coping with stress or managing fatigue. The study investigated how students’ interactions with VPs influenced learning and practice of NTS.

### Research question

The question underpinning this research was: How do interactions with VPs influence learning NTS in first and third-year undergraduate nursing students?

## Method

### The virtual simulated patient resource

The Virtual Simulated Patient Resource (VSPR) (www.vspr.net.au. Melbourne, Victoria. Australia) is a freely available web-based resource, funded by Health Workforce Australia and the Department of Health, Victoria, Australia. The primary author led the design, development and authorship of the VSPR. The resource uses e-Learning modules and branching narrative VPs to develop knowledge, skills, attitudes and practice of all categories of NTS, including communication, situation awareness, teamwork, leadership, decision-making, coping with stress or managing fatigue in undergraduate health professionals [6]. The study investigated how students’ interactions with VPs influenced learning and practice of NTS.

Grounded in constructivist theory, interactions with VPs support construction of new knowledge and understanding by interrogating prior knowledge and experience [7]. Students engaged in the simulation as themselves. The narrative VP used a “choose your own adventure game” approach with short video vignettes, depicting a patient’s story over time. The simulation progressed when students selected from two choices appearing on the screen following the video vignette. A decision tree using a branching algorithm determined the next video in the simulation sequence. Consequences of decisions resulted in positive or negative effects on the patient’s outcomes, providing intrinsic feedback to students on their actions. VP interactions were completed as independent and/or small group activities. In small group activities, learning was supported by group discussion at the end of each video vignette and a concluding facilitator-guided debriefing. Students have unrestricted access to the modules and VPs in the VSPR enabling repetition. Seven VP scenarios are available in the VSPR (Table 1).

**Table 1:** Outline of VP scenarios in VSPR

VP title	VP topic	Positive patient outcomes	Negative patient outcomes.	Target learner audience
Falls	Preventing patient falls in the clinical practice setting.	Patient assisted to toilet. Patient discharged 2 days later.	Patient falls when toileting independently. Sustains laceration to forehead, fractured right neck of femur and head injury. Patient remained in hospital for several weeks for treatment and rehabilitation.	Novice learner
Ward round	Interprofessional teamwork including patient as a member of the team.	Correct diagnosis and management and patient commenced on appropriate medication. Patient discharged 3 days later.	Incorrect diagnosis and management. Patient experiences acute pulmonary oedema and requires emergency medical team intervention. Patient admitted to high-dependency unit and follow-up rehabilitation for several weeks.	Advanced beginner
Aggressive patients	Recognising and responding to aggression.	Patient frustration and agitation correctly identified and managed resulting in de-escalation of the situation.	Patient frustration and agitation is not recognised. Patient behaviour escalates into aggressive violent outburst. Security measures are required to manage patient situation.	Advanced beginner
Community	Managing warfarin therapy in the community post coronary artery bypass grafts.	Patient receiving warfarin therapy has high International Normalised Ratio identified and emergency medical treatment instigated.	High international normalised ratio not identified. Patient sent home from clinic with no emergency treatment. Patient experiences large cerebral haemorrhage and dies shortly after.	Advanced beginner
Administering blood products	Risks associated with administering blood products in the clinical setting.	Incorrect patient identification is recognised. Correct patient is located and verified. Blood product administration proceeds without incident.	Following incorrect patient identification, wrong patient administered blood product. Patient experiences severe haemolytic transfusion reaction and admitted to intensive care unit.	Advanced beginner
Post-operative	Recognising and responding to patient deterioration in the immediate post-operative period.	Patterns and trends are recognised early with appropriate treatment implemented. Normal fluid balance returned in post-operative phase.	Patterns and trends are not recognised and no treatment implemented. Patient experiences acute kidney injury post-operatively.	Competent
Midwifery	Woman with epidural analgesia in labour experiences episode of hypotension.	A pathological fetal heart rate pattern is detected following episode of hypotension. Fluid resuscitation is initiated and a change to maternal position. Normal vaginal birth.	There is a delay in recognising, reporting and responding to a pathological fetal heart rate pattern. Fetal compromise ensues resulting in emergency caesarean section and neonate resuscitation.	Competent

### Design

This paper reports part of a larger multisite exploratory, qualitative research project using case study methodology [8] with focus groups and individual interviews. We were intent on insight, discovery and interpretation of the learning experience participants have when engaging with a particular case, in this instance, the VPs in the VSPR [9]. The year level of the undergraduate nursing student comprised the unit of analysis. The themes identified in Peddle et al. [4] provided the conceptual organisation for this case study, identifying areas of needed understanding, directing data collection and guiding interpretations [10]. University Human Ethics Research Committee granted ethical approval (Ethics approval ID number: CF12/3958 – 20120018910). Reciprocal ethical approval was obtained at all study sites.

### Population and sample

Purposive convenience sampling was used to identify suitable participants to address the research question. First- and third-year undergraduate nursing students from two university nursing schools in Victoria, Australia, using VSPR were invited to participate. Recruitment comprised either invitations disseminated through online subject forums or verbally after simulation activities. The primary author (MP) undertook all recruitment from March to April 2017. Forty-five first-year and 31 third-year students consented to participate.

### Data collection

A topic guide supported data collection via focus groups and individual interviews. The topic guide was piloted for acceptability, usability and question clarity, with outcomes guiding amendments to the question schedule and approaches (Additional file 1). Data collection occurred in person, in a private room, except for one focus group that used video-conferencing to a regional location. The primary author (MP) facilitated all focus groups and interviews except one, which due to scheduling issues was facilitated by another faculty member using the same topic guide. All focus groups and interviews were audio-recorded and professionally transcribed. In accordance with ethics approval, informed written consent was obtained prior to participation.

### Data analysis

Framework analysis supported by a codebook was used to analyse data. Framework analysis is appropriate in deductive, inductive or combined qualitative analysis [11], as it assists in organising and managing text so it can be systematically compared [11] and interpreted [12].

Themes identified in an integrative review by Peddle et al. [4] were used as theory driven, a priori codes in the codebook, guiding deductive data analysis. Two authors (MB and MP) independently coded a focus group transcript to test applicability of the codebook. Results were compared and modifications made to the codebook. Inductive data analysis enabled recognition of data-driven codes. Each code was discussed and added to the codebook by agreement. All codes in the codebook were given labels, defined with reference to existing literature and provided a description [12].

Codes were entered as nodes in NVivo (QSR International Pty Ltd. Melbourne, Victoria, Australia, Version 11), and one author (MP) coded all subsequent transcripts (*n* = 18). During the coding process, one author (MP) exported data from randomly selected nodes, which were examined by an additional author (MB) for coherency, consistency and fit. Specific areas of concern were discussed, leading to codes being reorganised and retitled on consensus. An extended summary of each code was developed, with illustrative quotes selected from the data from different units of analysis (year level of students). Codes along with associated illustrative quotes were reviewed, revised and grouped.

### Findings

One individual interview and ten focus groups were conducted with first-year students, and one interview and six focus groups with third-year students. First-year focus groups and the interview ranged from 14 to 37 min, with third-year focus groups and interview ranging from 20 to 53 min. Individual interviews were conducted where only one student accepted the invitation to participate but could not reschedule and wanted their voice to be heard in the research.

The unit of analysis in this case study was the year level of the student. An important finding highlighted by all students was that prior experience—as indicated by the year level—altered perception of cues and information in scenarios and affected options selected. Data indicated that first-year students, with limited or no prior experience in healthcare, experienced challenges with terms, language and situations portrayed complicating learning. On the other hand, third-year students conveyed that their prior experience enabled them to readily distinguish significant cues and important information, enabling them to perceive the care situation more easily.

Four themes were recognised in the data: how the VP enabled learning NTS, learning surrounding the VP encounter, changing the way students perceive practice and potential limitations to learning (Table 2).

**Table 2:** Themes, categories and sub-categories of student experiences interacting with the VPs

Theme	Category	Sub-category
1. How the VP enables learning to happen	Socialisation to role	*“This is how I should be.”*
		*“Develops self-confidence.”*
	Authenticity	*“Real but not real.”*
		*“The same thing happens.”*
		*“Sometimes bad things happen”*
	Learning through mistakes	
	Vicarious learning	
	Design	
2. Learning surrounding the VP encounter	“We all got to work together.” Reflective practice. “Connects with the content.”	
3. Changing the way students perceive practice	“We’re all involved in patient safety”. Practical value “That’s not the way we do it here!”	
4. Potential limitations to learning	Fear Overconfidence Barrier of the classroom Confusion Groupthink	

#### How the VP enabled learning NTS

Five categories describe how interactions with VPs facilitated learning NTS: socialisation to role, learning through making mistakes, vicarious learning, authenticity and design. The first two categories have multiple sub-categories.

#### Socialisation to role

The ‘socialisation to role’ category explores how the student came to understand the norms, expectations and behaviours expected in practice. There are two sub-categories: “this is how I should be” and “develops self-confidence”.

##### “This is how I should be”

All students articulated that interacting with VPs enabled them to think and feel like nurses: “It’s really making me see, putting myself in them and this is going to be me, this is what I’ve got to do, this is how it’s going to be, this is the relationship I need to have” (Yr1/Grp11/Participant 3). Students developed insight into the environment, characters and events that may occur during practice, enabling identification of appropriate behaviours in specific situations: “It just gives you examples of how things might go…” (Yr3/Grp1/Participant1). Interactions with VPs enabled students to value their role, demonstrated application of professional standards in practice and developed insight into working in interprofessional teams: “… it places you in a situation where it’s not just the patient. It’s putting you in situations like - staff members and doctors...” (Yr3/Grp4/Participant 5). Interactions with VPs enabled recognition of at-risk situations and behaviours and strategies to ask for help: “…it gives an example of the best way to… go to a buddy nurse and ask for help” (Yr3/Grp1/Participant 3). All students described how interacting with VPs role-modelled professional practice.

However, whilst some first-year students expressed that interacting with VPs validated preconceived expectations, for others, the simulation provided opportunities to reframe or clarify misconceptions: “You go in thinking the doctors tell you what to do and give you a checklist on what you have to do and stuff. That’s just what everyone portrays a nurse is – [then] you have the realisation that it’s not at all” (Yr1/Grp10/Participant1). Additionally, first-year students highlighted role modelling of communication with patients as important, whereas third-year students appreciated role modelling of specific communication skills including set handover structures such as Introduction, Situation, Background, Assessment, Recommendation (ISBAR) speaking up and reporting patient deterioration.

##### “Develops self-confidence”

Both year levels indicated interacting with VPs developed confidence in their abilities to respond and manage practice situations: “I feel more confident in how to handle situations like that now. I feel a lot more prepared in handling a patient and [undertaking] risk assessments” (Yr1/Grp4/Participant1).

However, where first year students described using the scenarios for skill development, third-year students described how they used the scenarios as a point of reference to “benchmark” (Yr3/Grp2/Participant1), reflect on and assess their practice. Third year students qualified this enabled reinforcement or correction of knowledge and practice, developing self-confidence: “I always look at the nurse who’s doing it and I see the mistakes they are doing. And “oh, I can do better than that!” Sometimes I actually see stuff that I…[am] missing - then I’m actually self-correcting. That actually builds confidence” (Yr3/Grp5/Participant6).

#### Authenticity

The authenticity of the VP enabled students to see relevance of the VP interaction to their professional development as it represented real practice that was familiar. There are three sub-categories: “real but not real”; “the same thing happens”; and “sometimes bad things happen”.

##### “Real but not real”

Most students reported the VPs as ‘real’, with authentic characters, situations, environments and events that appealed to students, enabling immersion and engagement, motivating learning. Authenticity in the interaction heightened awareness that medical errors can happen and how errors occur in practice with competent, experienced professionals: “These are actually people who are already professionals; they are registered nurses, doctors” (Yr1/Grp5/Participant 1). Many students conveyed that interacting with VPs made them cognisant of the imperfect reality of clinical practice, stating it was valuable to see ‘real’ practice: “Not everybody is going to be like a perfect friendly robot. You’re going to have co-workers who are going to be difficult. You’re going to have people and patients who aren’t going to be always respectful” (Yr1/Grp10/Participant 2). Many students were surprised how quickly clinical scenarios changed, with this variability adding authenticity to the experience: “Just to keep in mind that anything could happen to the patient and [it] might escalate very quickly” (Yr3/Grp7/Participant 4). Though, students from both years qualified that they could tell the experience was not real: “Like it didn’t feel not real, it seems pretty realistic for the most part” (Yr1/Grp1/Participant1) “…but you just know it’s not real” (Yr3/Grp 3/Participant1).

##### “The same thing happened”

Students described the situations, events, performance of characters and responses from patients as relatable and familiar, endorsing the interaction as relevant and credible: “When I did my placement, the same thing happened. When doctors were doing rounds, they included physio and the nurse… having arguments, as well” (Yr3/Grp4/Participant2). Students identified the scenarios emphasised various roles, multiple points of interaction and multitasking present in patient care activities, accentuating genuine risks associated when working in a team: “It shows you how hard it is to kind of communicate when you’ve got 1,000 things going on” (Yr1/Grp11/Participant 2).

##### “Sometimes bad things happen”

Many students thought the depiction of poor patient outcomes necessary for learning: “I think it’s useful to demonstrate severe consequences because there’s a lot of risk involved and sometimes bad things will happen and I think it’s something that we need to be exposed to” (Yr1/Grp10/Participant2). Some students valued experiencing poor patient outcomes via simulation rather than in practice: “To know what the terrible repercussions will be and I’d rather do it on this” (Yr3/Grp1/Participant2). Students described how interacting with the VP highlighted the likelihood they may experience poor professional practice: “…you know that there would be nurses like that out there that…does her poor version of [practice]. It’s scary” (Yr3/Grp3/Participant2).

However, third-year students were able to qualify that the patient outcomes depicted in the VP scenarios were believable. Furthermore, third-year students valued how interactions deepened their understanding of negative patient outcomes related to poor NTS.

#### Learning through making mistakes

Learning through making mistakes afforded students opportunities to see consequences of actions, galvanising some into action and prompting thinking through decisions.

All students stated it was beneficial to experience consequences of decisions made in the scenario especially when they ‘got it wrong’ (Yr3/Grp1/Participant1) and could visualise poor patient outcomes: “I think it allows you to make mistakes without actually hurting someone and learning…” (Yr1/Grp6/Participant1). Many students deliberately selected incorrect options to see the negative consequences, reporting the experience galvanised them into action: “It just shows that if you don’t speak up - what can come of it. It gives you the courage to speak up” (Yr3/Grp4/Participant7). Students appreciated understanding how mistakes and errors are managed: “That was quite a big eye opener. There are consequences for your patient [from]… mistakes but also their family as well and you’ve got to be the one to stand up and take responsibility for it” (Yr1/Grp10/Participant3).

Students from all year levels identified how interacting with VPs “really made you think” (Yr1/Grp3/Participant3) about decisions and potential consequences: “We got one of the questions wrong… Then it was like ‘oh my gosh, we didn’t even realise! We’re just like “Yes that sounds right”. We didn’t really think about it” (Yr1/Grp3/Participant2). Students clarified the interaction got them thinking ahead trying to predict outcomes: “Like you’re really thinking ahead and your sort of thinking what can go wrong or…how could this improve for the patient situation” (Yr3/Grp2/Participant1).

#### Vicarious learning

Strong emotional responses triggered by interacting with VPs afforded students feeling present in the care interaction, experiencing professional behaviours and the emotional burden of practice.

Students valued opportunities to experience clinical situations without the situation happening to them, learning via experiences of others: “It’s giving you an insight into an experience you haven’t got” (Yr3/Grp3/Participant1) and preparing them for future practice situations: “…instead of…freezing in the moment you have kind of seen it before” (Yr3/Grp1/Participant1). Many indicated they were present, participating in care activities, controlling the situation with responsibility for the patient: “I feel like…we were in the scene; like you were there…I saw myself as that nurse and whatever she said, I said. And how the doctor spoke with her, I just saw myself in…her shoes” (Yr1/Grp2/Participant1). However, some students felt like observers, with a “birds-eye view” (Yr3/Grp7/Particpant3).

Interacting with VPs triggered emotions including frustration related to practitioner performance and choice, pride when positive patient outcomes were achieved, and guilt, terror and fear when negative patient outcomes eventuated. Students indicated interactions developed empathy with patients as well as health professionals: “It sort of puts us in her shoes and thinking what would I be doing next if I was this person” (Yr3/Grp3/Participant3).

However, only first-year students reported interactions with VPs enabled them to experience emotions they would likely experience in practice, giving insight into reactions and the emotional burden of practice: “…it also shows you the emotional burden you’re going to be taking on. If you make a mistake, it’s real… from one little mistake, by not communicating. The patient gets hurt so it’s real” (Yr1/Grp11/Participant2).

#### Design

The VP design, including the organisation and visualisation, were beneficial to learning, supporting interpretation and easier recall. The VPs were reported to be well organised, easy to navigate and aesthetically pleasing. Students liked the flow of the VPs with short, sharp, detailed videos complementing learning. Students appreciated the variation to teaching approaches in curricula, opportunities for repetition reinforcing learning and consistency afforded for all students across learning experiences. Most students reported the visual depiction of the VP a major benefit for learning, with videos easier to interpret and recall in practice and the information staying with them longer: “If I see a video and I see how it’s done and I see how you’re meant to act, how your tone of voice is; that for me works better. I need to visualise something to get a better understanding” (Yr1/Grp3/Participant2). Students stated the VPs were easy to engage with and appreciated “not [being] thrown in the deep end” (Yr3/Grp2/Participant1). Students preferred the real human reactions and interactions portrayed in the VPs over mannequin-based simulation, commenting: “the patient actually responds” (Yr1/Grp4/Participant1).

#### Learning surrounding the virtual patient encounter

Students identified that learning content and activities surrounding the VP interaction supported learning knowledge, skill, attitudes and practice of NTS. There were three sub-categories: “we’ve all got to work together”, “reflective practice” and “connects with content”.

##### “We all got to work together”

Students reported learning in small group approaches was powerful. They valued group discussions enabling them to identify missed cues, view situations differently and clarify concepts: "if you watch it by yourself you don’t realise it but you’re focusing on one thing, but…when you start talking, everyone will go, “Didn’t you see that guy do this?” and, “What about when she did that?” And I’m like, “Oh, I didn’t even see that” (Yr3/Grp7/Participant3). Students valued opportunities to practise NTS including, appreciating other perspectives, managing conflict, considering different ways of thinking and collaborating with others to solve problems: “I like how the video made a classroom full of people with different skills work together… that’s what’s going to happen when we’re actually in the healthcare industry. So, it was good practice communicating and working with different people” (Yr1/Grp3/Participant2).

##### “Reflective practice”

Interacting with VPs promoted student reflection on practice: “It’s very good for reflective practice, learning what you’ve done, where you went wrong, how you’d improve it next time, why you shouldn’t have done that” (Yr1/Grp8/Participant3). Students across all year levels clarified they would reflect on their interactions with the VP and complete follow-up activities to make sense of learning. Other students explained they would repeat the interaction when they needed a refresher.

##### “Connects with the content”

Students reported interacting with VPs reinforced concepts learnt in other subjects. Students clarified the interaction offered more detail and a means to integrate learning as a cohesive whole: “I feel like it does connect with the content that we’ve been learning so far. I wasn’t thinking in my head ‘I don’t need to watch this, this is not relevant’. I was thinking ‘this is really important and I should use this resource’…even when I was doing lab, I felt like it was in touch with what my lab teacher was going through” (Yr1/Grp3/Participant1).

#### Changing the way students perceive practice

Students identified how interacting with VPs enabled application of specific strategies in practice. Three sub-categories were identified: practical value, “that’s not the way we do it here” and “we are all involved in patient safety”.

#### Practical value

Many students described application of skills learnt from interactions with VPs to situations in practice. Students clarified how the realistic presentation of situations in VPs made it easier to recall the scenarios in practice: “So, if you were in that same situation, you think back to this video it’ll be in your head” (Yr1/Grp8/Participant4). Many students described intentions to implement NTS demonstrated in VPs in their practice: “When you do watch it…you pick up little things, yes, that’s good…I’ll take that away with me. I like how she said that, I am [going to] use that” (Yr3/Grp1/Participant3). Students reported using reflective practices developed to reflect on their practice: “just to think after your working day, what I could do better, what went wrong and why it wrong and just thinking what can you improve” (Yr1/Grp5/Participant1).

However, first-year students identified benefits to technical aspects of practice such as one first-year student describing how participating in the VPs enabled her to manage a resident who had a fall. While third-year students identified how interactions shaped unconscious patterning: “It’s like we indirectly take stuff from them…I don’t go on placement and say I am [going to] do exactly this that I saw in the video. Then you do it and you realise… ok…I learnt that from the video” (Yr3/Grp1/Participant3).

##### “That’s not the way we do it here!”

All students reported discord when transferring NTS learnt interacting with VPs to the clinical setting several weeks after the activity. A first-year student described: “Placement was a bit confronting for me it was…not what I expected. We had been taught a lot of – ‘This is the way things should go.’ But…in the placement things did not necessarily go like that. I think that perhaps not all the things we learnt at the university were in place in the workplace” (Yr1/Grp1/Participant2). Third-year students described shortcuts taken, practice not evidence-based and gaps in knowledge regarding NTS.

##### “We’re all involved in patient safety”

Students reported interacting with VPs assisted understanding the link between NTS and patient safety: “The point is that…it doesn’t matter what kind of level you are…we’re all involved in a patient’s safety.” (Yr1/Grp5/Participant1).

However, most first-year students were not aware of, or did not have, clarity around NTS in practice. Conversely, most third-year students articulated clear understanding of NTS: “It could be fatal on the patient’s outcome, as well. It could lead to either further complications, or it could lead to death of the patient” (Yr3/Grp4/Participant2).

### Potential limitations to learning

Five categories were associated with potential limitations to learning including: fear, overconfidence, barrier of the classroom, confusion and groupthink.

#### Fear

Only first-year students raised the potential negative impacts of poor patient outcomes on learning. First-year students were surprised by “all the bad outcomes” (Yr1/Grp5/Participant1), expressing fear and being “freaked-out” (Yr1/Grp5/Participant1). These students suggested portrayal of poor patient outcomes might cause overthinking, hesitation or second-guessing oneself in practice.

#### Overconfidence

Only first-year students indicated that interactions with VPs had corrected overconfidence: “… I thought I would not make the patient fall. That’s it, I’ve got it! And then when I’m making the choices I’m like, oh okay” (Yr1/Grp8/Participant4). Conversely, third-year students highlighted risks in developing overconfidence following the VP interaction: “You get confidence but not real confidence” (Yr3/Grp3/Participant2) and "I look at all the procedures and I’m like, “Yeah, I can do this.” (Yr3/Grp5/Participant6). Other third-year students indicated: “Like if it was an emergency and they made you go to the phone, you wouldn’t be so confident, but…it wouldn’t phase you” (Yr3/Grp3/Participant2).

#### Barrier of the classroom

Some students from both year levels could not move beyond the barrier of the classroom environment and engage in the learning: “I could tell like it’s sort of set up, just like the way people communicate. Like they know what they’re going to say, which doesn’t really happen in real life, but the situation was real, but I could just tell that it wasn’t real” (Yr1/Grp4/Participant 2). Some qualified they were waiting and expecting something to happen making the experience feel contrived. One student clarified that to consolidate learning achieved from interacting with VPs, they would need to experience the situation in practice.

#### Confusion

Several students across both year levels reported they found some options in the VP interaction confusing, with wording difficult to understand such as “Vital signs within reportable limits” and “Patient observations demonstrate a significant pattern.” However, nearly all students qualified; once the next video in the VP sequence played, they could make sense of the question.

However, first-year students reported issues with fundamental medical terminology and understanding clinical features of the scenario, whereas third-year students expressed frustration at limited options available: “With the options, I thought it was limited, because you only had two choices. I thought, if there was maybe…four choices, at least you were more able to…critically think a bit more” (Yr3/Grp4/Participant9), clarifying choices appeared “black and white” (Yr3/FG5/Participant2) with no right options available at times.

#### Groupthink

Students indicated it was a “majority rules” (Yr1/Grp6/Particpant1) approach and communicated at times “there was a bit of tension in the class with the other students” (Yr1/FG9/Participant2) and discussions “kind of turned into an argument” (Yr1/FG7/Participant2), with “people being disrespectful” (Yr1/FG7/Participant1) due to dissenting opinions. At times, when "somebody brought up one point we all kind of changed our minds’ (Yr1/Grp9/Participant1), others chose to remain quiet: “I picked the correct answer first but then when everyone else said it was the other one I was kind of like okay I’ll kind of be a bit quiet” (Yr1/Grp8/Participant5) and conform to groupthink.

## Discussion

This research reinforces findings indicating interactions with VPs develop knowledge, skills, attitudes of NTS in undergraduate health professionals via socialisation to professional role and authenticity in the VP interaction, with skills transferrable to practice. It clarifies that learning NTS via VP interactions can change the way students perceive practice. New themes in this research suggest learning through making mistakes, vicarious learning, design of the VP and learning surrounding the VP interaction are significant factors facilitating learning NTS via VP interactions. Lastly, this research highlighted potential limitations to learning and practise of NTS including fear, overconfidence, classroom barrier, confusion and groupthink.

A number of particularly interesting findings from this research add to existing understandings of VPs. These are learning through mistakes, vicarious learning, groupthink, impact of overconfidence on practice and consideration of the prior experience of the learner. Each of these findings is discussed in turn.

Learning through mistakes is proposed to have a powerful impact on learning [13]. When learners make mistakes, they have strong emotional responses, which may encourage, motivate [13–15] and promote deep learning, supporting knowledge retention and transfer of learning [13]. Additionally, simulated mistakes can amplify student awareness of the possibility of clinical errors in practice [15, 16]. The design of the VP in this study, to demonstrate negative patient outcomes, supported discussion, developing critical thinking [17] and identification of gaps in knowledge and skill [13]. Students reported that experiencing and exploring mistakes in interactions with VPs facilitated learning. They described strong emotional reactions to negative outcomes such as guilt, terror and fear, as well as sometimes deliberately seeking to make mistakes to see what the consequences were. This led to them to reflect on how they would act in practice and seeing patient safety as everyone’s responsibility.

Conversely, this study highlighted that learning through mistakes could negatively influence learning, causing students to be scared or “freak-out” about possibilities of harming patients in practice. Experiencing negative consequences can be traumatic for some learners, leading to defensive practice [18] including fear of failure in practice and hesitation in initiating patient care [19]. In this research, whilst interaction with the VP made real for learners the possibilities of making mistakes in practice, they also sparked feelings of guilt, terror and fear. When VPs depict error and adverse events resulting in serious harm or death to patients, consideration may be needed by faculty to provide opportunities for reflection. Reflection can enable re-contextualisation of the experience and facilitation of learning [18] to translate the experience of “getting it wrong” into positive learning about NTS.

Vicarious learning proposes it is possible to learn through another’s first-hand experience [20]. Vicarious learning is advocated to be an important strategy for novice learners who have a deficit of prior knowledge and experience [21]. Timbrell [21] suggests vicarious learning assists novices to generate personal meanings from secondhand experiences, supporting transfer of learning to clinical practice [21]. In this research, students indicated interactions with VPs placed them in the clinical situation, involving them in the patient care situation, enabling them to contemplate how they may think, respond and act in future clinical situations. Additionally, vicarious learning enables coding of observed behaviours to be used to guide future action [22]. In this study, students highlighted interactions with VPs supported framing future behaviours, “I’m going to use that”, to guide practice.

Novice learners benefit from small group discussions as they provide opportunities for learners to recognise important factors and cues, connect knowledge and assess and analyse their own understanding [23]. However, this research shows a potential limitation to learning NTS is “groupthink”. Groupthink is when humans influence or bias each other’s decision-making [24]. In this research, learners reported exactly this. Groupthink can result in poorer decisions, as members of the group may not consider all options, or some group members may impose self-censorship to avoid inconsistency [24]. If learners perceive an environment as psychologically safe, they are more likely to engage in open discussion and share ideas without fear of repercussions [25]. Strategies to facilitate this safe environment where participation and learning is enhanced and disengagement is minimised [26] in interactions with VPs warrants further exploration.

Improved self-confidence is often cited as an outcome from simulation-based learning [27]. This research identifies that there is also a risk of developing overconfidence, or “miscalibration of one’s own sense of accuracy and actual accuracy” [28]. Overconfident practitioners are deemed more likely to make mistakes [29] as they are less likely to “consult with colleagues or utilise tools, protocols or practice guidelines to aid their decision-making” [30]. Moreover, overconfidence is linked to diagnostic error [28]. Strategies to reduce risks of developing overconfidence include refining expertise via further education and training, reflective practice to increase self-awareness and constructive feedback to assist learners be better calibrated to their personal performance [28]. This study suggests a need for balance between reflection and constructive feedback to assist learners better align perceived competency with actual competency in interactions with VPs.

Other limitations to learning in VP interactions included frustration at the wording of options to progress the simulation and third-year students perceiving “no right” options available to respond to the clinical situation. This study highlighted that students’ prior experience influenced perception of cues and ability to interpret significance of information, which in turn affected options selected. As learners developed expertise, they moved from reliance on objective facts and rules to using past concrete experiences and seeing the situation as a whole, rather than a compilation of its parts [31].

First-year students are novice learners; hence, when wording and question formulation deviated from the rule bound principles, they experienced confusion and frustration [31]. These students possessed limited understanding of NTS and appreciated role modelling of specific behaviours in practice that assisted in clarifying misconceptions. However, lack of experience for first-year students may have created confusion, which at times detracted from their learning. Moreover, first-year students suggested that interactions with VPs that result in poor patient outcomes might develop fear of practice.

Accordingly, third-year students, as advanced beginners, were able to perceive the situation holistically and based on their prior concrete experiences, at times suitable options to respond to clinical situations were not available [31]. Third-year nursing students demonstrated clear understandings of NTS, using the believable, realistic, VP scenarios to benchmark and assess their own practice. However, these students indicated there were risks in developing overconfidence in practice and that at times, no right options were available to progress the simulation, due to their prior clinical experience.

### Strengths and limitations

Theoretical suppositions were used to guide data analysis as per case study methodology. Data collection occurred across 2-year levels at two distinct sites. Transferring findings to other student cohorts is supported by the naturalistic study setting, with multiple data sources. Themes, categories and sub-categories were created, labelled and defined by MP and MB by agreement. Data were examined for consistency, coherency and fit with conflicts resolved through discussion and consensus from all authors. Focus groups and interviews facilitated a detailed, in-depth understanding of the learning experience of undergraduate nursing students. However, caution must be exercised as focus group composition may not be representative of the wider population and there is risk that data arises from dominant participants. During data collection, the primary author was on research leave and had no formal relationship with the students who participated in the research. Moreover, it is important to note that the primary author led the design, development and authorship of the VSPR. Hence, the primary author worked closely with co-authors to ensure rigour in the research, transparency in analysis and trustworthiness of findings.

### Recommendations

The focus of this research was undergraduate nursing students. Additional research is required to examine the learning experience of students interacting with VP from other professions. Additionally, the role of faculty and debriefing in reconceptualising learning from negative patient outcomes, specific facilitation strategies to prevent groupthink, and the influence of reflection and constructive feedback on overconfidence warrant further investigation.

There were a number of possible recommendations for practice arising from this research. This study suggests that in order to facilitate learning NTS though VP interaction for undergraduate nursing students, faculty can provide opportunities for reflection, supported by constructive feedback, to enable learners to reconceptualise experiences and feelings into learning. Additionally, faculty are advised to be conscious of the potential for groupthink and have the ability to maintain awareness in the classroom, whilst enabling a supportive and secure learning environment. Finally, faculty can be mindful that the level of complexity of the learning in the VP interaction fits with the level of experience of the learner.

## Conclusion

Interactions with VPs in VSPR influence learning knowledge, attitudes and practice of NTS in undergraduate nursing students via authenticity in the VP interaction, socialisation to the professional role, vicarious learning and learning by making mistakes. This research highlights the importance of connection of the VP interaction to learning surrounding the activity to maximise learning outcomes. To manage potential limitations to learning from VP interactions including fear, overconfidence, groupthink and confusion; facilitation approaches, opportunities for reflection, constructive feedback and debriefing may be key. This study demonstrates learning NTS via interactions with VPs can change how students perceive practice, with learning transferable to the clinical setting to support safe and competent patient care.

## Additional file

Additional file 1 : Focus group and interview guide. (DOCX 13 kb)

## Abbreviations

ISBAR: Introduction, Situation, Background, Assessment, Recommendation
MB: Margaret Bearman
MP: Monica Peddle
NTS: Non-technical skill
VP: Virtual patient
VPs: Virtual patients
VSPR: Virtual Simulated Patient Resource
