# Supplementary material for: Exploring undergraduate nursing student interactions with virtual patients to develop ‘non-technical skills’ through case study methodology
Source: Adv Simul (Lond). 2019 Feb 13;4:2. doi: 10.1186/s41077-019-0088-7 (PMC6373120; doi:10.1186/s41077-019-0088-7)
Supplement: Supplementary file 1 — : Focus group and interview guide. (DOCX 13 kb) [file 41077_2019_88_MOESM1_ESM.docx]

Additional File 1. Focus group and interview guide.

**Introduction**

Now that you have undertaken the VSPR program, I’d like to ask you some questions. I encourage you to discuss with each other and share your experiences with the group. In a focus group, we are not seeking consensus but would like you to share your opinions with each other about the resources. Please be honest in your responses. I welcome positive and negative comments equally.

**Ground Rules** - only one person speaking at a time, no side conversations among neighbours, everyone participating with no one dominating, and so on.

VSPR is a web based resource that consists of six modules and five play your own adventure game type simulation scenarios. This resource explores that application of NTS including, teamwork, communication, leadership, situational awareness and decision making in health professional practice. The resource contains modules and scenarios. We will be exploring the scenarios today. (VISUALS)

| ICEBREAKER | Introduce each other around the table. |
| --- | --- |
| INTRODUCTORY QUESTIONS | 1. Let's start by having you identify everything that is involved, one way or another, with non-technical skills in healthcare/VSPR scenarios. Share your thoughts with each other. 2. What is your understanding of the role of non-technical skills in patient care? Share your thoughts with the group. |
| QUESTIONS | 1. What were your thoughts, feelings and perceptions at the time when working through the VSPR scenario? 2. How did VSPR scenarios contribute to your understanding, thinking or practice of NTS? Which component or scenario/s and how? If not, can you state why? 3. What was helpful about the VSPR scenario? Why? What was unhelpful? Why? 4. Thinking about your clinical placement, can you give an example of when you observed effective NTS at work? How did VSPR scenario learning affect this? 5. Did you face challenges with using NTS in your clinical placements? If so, which ones? Why? If not, why? 6. Having viewed the virtual simulated patient scenarios, was there a relation to the clinical setting? 7. What was most helpful for your clinical placements from VSPR scenario? Has anything been unhelpful for your clinical placements? 8. Did anything surprise you about the content of the VSPR? 9. Is there anything else that we should have included? |
| FINAL QUESTION | Ask each participant to give a final summary statement |
